# Commentary: What more can we learn from early learning theory? The contemporary relevance for behaviour change interventions

**DOI:** 10.3389/fpsyg.2018.00023

**Published:** 2018-01-31

**Authors:** Samuel Ginja

**Affiliations:** Institute of Health and Society, Newcastle University, Newcastle upon Tyne, United Kingdom

**Keywords:** learning theory, theory, behavior change, B. F. Skinner, cognitive science

Marie Johnston's “What more can we learn from early learning theory? The contemporary relevance for behaviour change interventions” (Johnston, 2016) is a good summary of key learning theory constructs, with specific examples of their application in health behavior change research. Early learning theory is introduced as a synonym of classical and operant conditioning theories and research, although the examples provided throughout the paper are mainly related with operant conditioning. The aims of behavioral science are described in the terms of the father of operant conditioning, B. F. Skinner, as being the prediction and control of behavior. Johnston goes on to say that learning theory-based, health-related interventions acquired prominence in the 1960s and 1970s and “focussed on behaviour while still allowing that cognitive processes might have a causal role” (Johnston, 2016, p. 1), citing A. Bandura (Bandura, 1969). She also states that the main intervention developments at that time were in cognitive theory and methods, although no references are provided for this claim, and that “subsequent explanations [in learning theory] frequently refer to cognitive processes that might explain stimulus control” (Johnston, 2016, p. 2). Thus, Johnston suggests that early learning theory was confined to classical and operant conditioning but later, and presumably current, learning theory included cognitive processes. Such description of how the scope of learning theory evolved may well be accurate, but it then seems that everything in psychology is synonym of learning theory.

This question may not be central to Johnston's paper but it is raised by it, and is of relevance to those who seek to understand the differences (or similarities) between the various theoretical approaches within psychology. In general, the behavioral principles she describes are clear and logically connected within a coherent body of research, which historically is associated with behaviorism. I am less sure the same could be said had her paper focused on principles of “later” learning theory. While it is relatively easy to understand concepts such as intermittent reinforcement, there seems to be less clarity in Sheeran et al.'s findings on “stimulus control processes involving association with implicit cognitive or affective processes without the need for conscious or implicit motivation” (as cited in Johnston, 2016, pp. 2–3). Similarly, there is an obvious and direct link between the Premack principle and the selection of reinforcement strategies based on people's reported favorite hobbies, but it is less obvious how cognitive terms have shaped the development of behavioral interventions (e.g., Ginja et al., 2017).

It is interesting to note that behaviorist B. F. Skinner, who is the most cited author in Johnston's paper and who brought to light many of the behavioral processes she describes, had always strongly opposed cognitive explanations. One point of disagreement seems particularly challenging to a reconciliation between behaviorists and cognitivists: for B. F. Skinner, and generally for behavior analysts, causes of behavior are to be found outside individuals, namely in their interactions with the environment (social and non-social), which include contingencies of reinforcement that result in learning (behavior change) during an individual's lifetime (e.g., speaking a language) and contingencies of survival which are responsible for our genetic predispositions (e.g., tendency to make vocal sounds). With cognitive models, which may or not take into account the effects of environment, processes such as thoughts, beliefs, and attitudes are said to play a causal role in behavior. As Johnston says, the predictability of behavior can be explained by the persistence of “causal factors such as thoughts or rewards” (Johnston, 2016, p. 2). On this point, B. F. Skinner's position is unambiguous: “[cognitive terms] are troublesome not because they raise questions about dimensions but because they assign the initiation of behaviour to the person rather than to that person's genetic and personal history” (Catania, 1988, p. 204). Because cognitive processes (which are different from physiological processes) cannot be directly measured or manipulated, he justifies the non-causality of cognitive events on the grounds of pragmatism: “Our only chance of solving our problems is to look at the variables of which our behaviour is a function rather than at the mental events which serve as current surrogates of those variables” (Catania, 1988, p. 273). For behavior analysts, the question is not whether strategies which are typically treated as being cognitive, or which were developed by cognitive researchers, work or not, but whether cognitive processes need to be hypothesized to account for their success. For example, the potential of motivational interviewing or of the therapeutic relationship to engender behavior change is widely accepted but it may be possible to explain how they work in terms of contingencies of reinforcement (Follette et al., 1996; Christopher and Dougher, 2009).

In sum, Johnston suggests that learning theory posits the existence and causality of cognitive processes in behavior, but the behavioristic discoveries justly highlighted in her paper were derived from a philosophy of science standing at sharp contrast with cognitivism. This raises the question of how we should consider the fundamental differences characteristic of each of the two movements which Johnston presents together, and what, if any, are the implications of such considerations to explaining and studying behavior.

## Author contributions

The author confirms being the sole contributor of this work and approved it for publication.

### Conflict of interest statement

The author declares that the research was conducted in the absence of any commercial or financial relationships that could be construed as a potential conflict of interest.

## References

[B1] Bandura A. (1969). Principles of Behavior Modification. New York, NY: Holt, Rinehart & Winston.

[B2] Catania A. C. (1988). The Selection of Behavior: The Operant Behaviorism of BF Skinner: Comments and Consequences. New York, NY: Cambridge University Press.

[B3] Christopher P. J. Dougher M. J. (2009). A behavior-analytic account of motivational interviewing. Behav. Anal. 32, 149–161. 10.1007/BF03392180 22478518 PMC2686983

[B4] Follette W. C. Naugle A. E. Callaghan G. M. (1996). A radical behavioral understanding of the therapeutic relationship in effecting change. Behav. Ther. 27, 623–641. 10.1016/S0005-7894(96)80047-5

[B5] Ginja S. Arnott B. Namdeo A. McColl E. (2017). Understanding active school travel through the Behavioural Ecological Model. Health Psychol. Rev. 12, 1–17. 10.1080/17437199.2017.1400394 29098932

[B6] Johnston M. (2016). What more can we learn from early learning theory? The contemporary relevance for behaviour change interventions. Br. J. Health Psychol. 21, 1–10. 10.1111/bjhp.12165 26482915

